# Peer review of "The Role of Ultrasound in Epicutaneo-Caval Catheter Insertion in Neonates: Systematic Review, Meta-Analysis and Future Perspectives"

_diagnostics, 2023, doi:10.3390/diagnostics13172850_

Round 1

Reviewer 1 Report

Dear authors,

Indeed, a very interesting topic and well-presented review. I want to congratulate on the way you have conducted this systematic review; I appreciate you have included PRISMA criteria (figure 1).

Additionally, I would like you to improve the introduction and the abstract presentation.

Also, figures 2,3,4 are illegible. Please replace them or adjust quality if possible.

I think it would be better if you could integrate the figures into the manuscript. Personally , I think that a separate section for figures cuts the flow of the manuscript. Please refer in the text for figures 2,3,4,5.

Did you get permission to include the protocol of G Barone et al to your manuscript?

Please remove 6.Patents.

  1.  

English is ok, minor corrections.

Author Response

Additionally, I would like you to improve the introduction and the abstract presentation.

The abstract and the the introduction were improved as requested

Also, figures 2,3,4 are illegible. Please replace them or adjust quality if possible.

Thank you for your comment; we apologize for the inconvenience, we changed the figure.

I think it would be better if you could integrate the figures into the manuscript. Personally, I think that a separate section for figures cuts the flow of the manuscript. Please refer in the text for figures 2,3,4,5.

Thank you for your suggestion. The layout sent to the reviewer is decided by the journal. In the final version of the manuscript the figures will be integrated in the manuscript as suggested.

Did you get permission to include the protocol of G Barone et al to your manuscript?

Thank you for your questions. We have permission to include the Neo echo tip and also G Barone is an author of the present manuscript

Please remove 6.Patents.

Thank you, we removed patents in the text

Reviewer 2 Report

This paper shows an exhaustive metanalysis of the US vs. Xray to localize i.v. lines in newborns. This is a huge problem in the approach to newborns’ care in NICU, and the paper offers some  useful insights. This article can be accepted. The Authors should check for CAJ abbreviation.

Author Response

This paper shows an exhaustive metanalysis of the US vs. Xray to localize i.v. lines in newborns. This is a huge problem in the approach to newborns’ care in NICU, and the paper offers some  useful insights.

This article can be accepted.

Thank you for your comment.

The Authors should check for CAJ abbreviation.

Thank you for your suggestion. We added the abbreviation.

Reviewer 3 Report

Dear Editor

The manuscript explored
the role of ultrasound in epicutaneo-cava catheter insertion in neonates: systematic review, meta-analysis and future perspectives. My overall evaluation of the manuscript is positive. There are a number of minor revisions, formal and scientific aspects that should be addressed.

1.      It is necessary for the authors to specify exactly which month from 2022 the articles have been collected.

2.      It is necessary to explain how to select search keys in the materials and methods section.

3.      Regarding Figures 3, 4, 5, and 6, the writing features such as using capital letters at the beginning of the sentence are not observed.

4.      It is necessary to explain more about figures 2 and 4 in the discussion section.

5.      The discussion section is not well written and needs to be rewritten. Especially about which differences are statistically significant at what level, explanations should be given.

In some parts, especially the subtitles of some figures, the principles of writing have not been observed.

Author Response

 The manuscript explored the role of ultrasound in epicutaneo-cava catheter insertion in neonates: systematic review, meta-analysis and future perspectives. My overall evaluation of the manuscript is positive. There are a number of minor revisions, formal and scientific aspects that should be addressed.

  1. It is necessary for the authors to specify exactly which month from 2022 the articles have been collected.

Thank you this information was added in the paper

  1. It is necessary to explain how to select search keys in the materials and methods section.

Thank you for your comment. Unfortunately, I am not able to reply since I did not understand clearly the issue. Could you please rephrase your observation?

  1. Regarding Figures 3, 4, 5, and 6, the writing features such as using capital letters at the beginning of the sentence are not observed.

Thank you for your comment, the changes were made

  1. It is necessary to explain more about figures 2 and 4 in the discussion section.

Thank you for your comment, the discussion was changed

  1. The discussion section is not well written and needs to be rewritten. Especially about which differences are statistically significant at what level, explanations should be given.

The discussion was improved

Reviewer 4 Report

The article The role of ultrasound in epicutaneo-cava catheter insertion in 2 neonates: systematic review, meta-analysis and future perspec- 3 tives“ presented review of metaanalysis of the NICU intervention. The presenters presented the given objectives of the work in an adequate way, with excellent graphic, pictorial and numerical representations, and comments in the components of the article, along with a discussion of similar research and citations of adequate fresh literature. In this form, I have no objections to the components of the work, and I suggest it for publication in a journal.

Author Response

The article The role of ultrasound in epicutaneo-cava catheter insertion in 2 neonates: systematic review, meta-analysis and future perspec- 3 tives“ presented review of metaanalysis of the NICU intervention. The presenters presented the given objectives of the work in an adequate way, with excellent graphic, pictorial and numerical representations, and comments in the components of the article, along with a discussion of similar research and citations of adequate fresh literature. In this form, I have no objections to the components of the work, and I suggest it for publication in a journal.

Thank you for your comment.

Reviewer 5 Report

The authors present a review article on the topic of the role of ultrasound in epicutaneo-cava catheter insertion in neonates. The authors performed a systematic review and a meta-analysis of this topic.

The article discusses and important topic. US is a radiation-free technique that can be of great assistance in evaluation of catheter tip and its use in children should be promoted. However, scientific work also needs to be needs rigorously evaluated so that we can have no doubts regarding the presented results. 

I have some more detailed comments regarding this paper.

The authors, according to the title, intend to specifically discuss epicutaneo-cava catheter insertion (ECC). In the article, it is not described what ECCs exactly are and how they differ from the more known peripherally inserted central catheters (PICC). According to the search terms reported, in the analysis that they performed, it seems that the whole group of PICCs was included however. Therefore, it would be appropriate to change the title of the article instead of ECC to PICC, and everywhere else. Currently, the separation of the two terms is only confusing the readers.

Page 2, line 56: »Currently, chest/abdominal radiography (X-ray) remains the most common technique to determine tip position of ECC but unfortunately is not accurate, is a post-procedural methodology, and exposes neonates to ionizing radiation.« The statement that radiography is not an accurate method for determining catheter tip position is too dramatic and simply false. Indeed, there are limitations to radiography, including sometimes poor visualization of the catheter, which hasn't been stated there, but it is an accurate method that has been the standard of clinical care for decades!

Page 2, line 92: »The aim of the present paper is to perform a systematic review and meta-analysis about tip location of ECCs and give the readers a focus on future research.« The aim of the article is not specific enough, even false. Your aim was not to determine the tip locations of ECCs, but to assess the methods to evaluate its location. Please be exact, as the aim of the paper is one of the most important parts.

Page 3, line 120: Results: Firstly, a more detailed flow-chart that in detail explains the database search should be included. Explain exact search terms, what were results from each database, since you included more. Who performed the search. Who performed the title/abstract reading – were it multiple researchers? How did they corelate? Were 91 articles read in total or only the abstract? How were the 200 excluded? Currently, this is not very clear. In the review articles, it is extremely important to get the detailed search process, including the dates of the search, with all primary results and excluded articles. 

Page 6, line 203: Discussion: First part of discussion is exact repetition of the introduction. The discussion part should begin by discussing the findings of the authors study and then later put that into context by comparing it to previous studies/reports. 

Page 7, line 226: »In this Meta-analysis, the percentage of agreement between US and X-ray is 89%, this data is in line with what have been reported in the literature« This wording is very awkward. You analyzed this »literature«, it is expected that your results are in line with the same source that you analyzed.

Page 7, line 229: »However, this raw data about the performance of US is for sure underpowered for the reasons mentioned early: in most of the studies included there is no standardized protocol and there is no mention about the training for all healthcare providers in visualizing the tip of the catheter using US . These two issues are for sure very relevant in establishing the US performance, in fact several studies have proved a direct relationship between the number of tips visualized and the training of the operators.« Regarding »for sure« and the opinion of the authors, unfortunately this is not a scientific method that should have a place in a review article. One must note that typically training of neonatologists regarding the interpretation of radiography is also limited.

Page 8, line 288: »Even with this important limitation in this metanalysis the ability to detect the tip location in a correct, central position is 87% for US and only 77% for standard X-ray, with RR 0.74 [0.60 – _0.90], p=0.002. These data prove that US is for sure a more specific and accurate method for the study of a central tip position than standard radiography and it high likely that this data can be improved with training and a solid tip location protocol.« Again »for sure« and authors opinion... including specificity and accuracy (were both analyzed?) – which do the reported numbers represent? Even when these numbers are accurate and show that US can be a superior technique, it is important to highlight that doctors need sufficient training to be able to have such success with US; this has to be included in such concluding statements.

Page 8, line 293: I don't understand the relevance of the whole Neo-ECHOTIP protocol and the whole page dedicated to it.

Regarding conclusion, again, I think it is extremely important to highlight the need for US training and experience with its use to achieve good results.

Should be improved.

Author Response

The authors present a review article on the topic of the role of ultrasound in epicutaneo-cava catheter insertion in neonates. The authors performed a systematic review and a meta-analysis of this topic. The article discusses and important topic. US is a radiation-free technique that can be of great assistance in evaluation of catheter tip and its use in children should be promoted. However, scientific work also needs to be needs rigorously evaluated so that we can have no doubts regarding the presented results.

Thank your for your comment. This is not the purpose of our manuscript. We performed a systematic review and meta-analysis according to the PRISMA (Preferred Reporting Items for Systematic reviews and Meta-analysis). We did not report any single issue of the selected papers this would require a different approach since we must aknowledge that every papers published has got its methodogical issues and limitation.

However even the raw data support the use of US for tip location and this is in-line with all the current guidelines for vascular access

I have some more detailed comments regarding this paper.

The authors, according to the title, intend to specifically discuss epicutaneo-cava catheter insertion (ECC). In the article, it is not described what ECCs exactly are and how they differ from the more known peripherally inserted central catheters (PICC). According to the search terms reported, in the analysis that they performed, it seems that the whole group of PICCs was included however. Therefore, it would be appropriate to change the title of the article instead of ECC to PICC, and everywhere else. Currently, the separation of the two terms is only confusing the readers.

The title was not changed however we added in the introduction and in the discussion a clear and robust explanation for the use of the word ECC, we also explained the reason for using the word PICC

Page 2, line 56: »Currently, chest/abdominal radiography (X-ray) remains the most common technique to determine tip position of ECC but unfortunately is not accurate, is a post-procedural methodology, and exposes neonates to ionizing radiation.« The statement that radiography is not an accurate method for determining catheter tip position is too dramatic and simply false. Indeed, there are limitations to radiography, including sometimes poor visualization of the catheter, which hasn't been stated there, but it is an accurate method that has been the standard of clinical care for decades!

Thank you for comment. We have been wrong for decades! Please refer to the current INS guideline for tip location. It’s clearly stated that conventional radiology is NOT accurate. This evidence is not only supported in data availble in newborn but also in adult and children.

Page 2, line 92: »The aim of the present paper is to perform a systematic review and meta-analysis about tip location of ECCs and give the readers a focus on future research.« The aim of the article is not specific enough, even false. Your aim was not to determine the tip locations of ECCs, but to assess the methods to evaluate its location. Please be exact, as the aim of the paper is one of the most important parts.

Thank you for your comment. This review is indeed focused on the method of tip location. The aim was changed as requested

Page 3, line 120: Results: Firstly, a more detailed flow-chart that in detail explains the database search should be included. Explain exact search terms, what were results from each database, since you included more. Who performed the search. Who performed the title/abstract reading – were it multiple researchers? How did they corelate? Were 91 articles read in total or only the abstract? How were the 200 excluded? Currently, this is not very clear. In the review articles, it is extremely important to get the detailed search process, including the dates of the search, with all primary results and excluded articles. 

Thank you for your comment the results were changed accordingly

Page 6, line 203: Discussion: First part of discussion is exact repetition of the introduction. The discussion part should begin by discussing the findings of the authors study and then later put that into context by comparing it to previous studies/reports. 

The discussion was changed

Page 7, line 226: »In this Meta-analysis, the percentage of agreement between US and X-ray is 89%, this data is in line with what have been reported in the literature« This wording is very awkward. You analyzed this »literature«, it is expected that your results are in line with the same source that you analyzed.

Thank you for your comment. The text was changed

Page 7, line 229: »However, this raw data about the performance of US is for sure underpowered for the reasons mentioned early: in most of the studies included there is no standardized protocol and there is no mention about the training for all healthcare providers in visualizing the tip of the catheter using US . These two issues are for sure very relevant in establishing the US performance, in fact several studies have proved a direct relationship between the number of tips visualized and the training of the operators.« Regarding »for sure« and the opinion of the authors, unfortunately this is not a scientific method that should have a place in a review article. One must note that typically training of neonatologists regarding the interpretation of radiography is also limited.

Thank you for your comment. “For sure” was replaced. However the training of neonatologists regarding the interpretation of radiography is also limited is not supported by any data to the best of our knowledge. Therefore it is reasonable to make assumption on this issue

Page 8, line 288: »Even with this important limitation in this metanalysis the ability to detect the tip location in a correct, central position is 87% for US and only 77% for standard X-ray, with RR 0.74 [0.60 – _0.90], p=0.002. These data prove that US is for sure a more specific and accurate method for the study of a central tip position than standard radiography and it high likely that this data can be improved with training and a solid tip location protocol.« Again »for sure« and authors opinion... including specificity and accuracy (were both analyzed?) – which do the reported numbers represent? Even when these numbers are accurate and show that US can be a superior technique, it is important to highlight that doctors need sufficient training to be able to have such success with US; this has to be included in such concluding statements.

Thank you for your comment. The sentence was rephrased. We did underline the vital role of the traning in the whole paper

Page 8, line 293: I don't understand the relevance of the whole Neo-ECHOTIP protocol and the whole page dedicated to it.

This is in contrast with your previous statement. The use of a standardized protocol is the first step to train the operators and give them instruments to effectively perform tip location.

Regarding conclusion, again, I think it is extremely important to highlight the need for US training and experience with its use to achieve good results.

In the conclusion we already stated that the role of training is crucial which means that is very important

Reviewer 6 Report

Thank you for requesting  to provide a review of this article, about the role of the ultrasound in evaluating the epicutaneo-caval catheter insertion in neonates.

   The main purpose of the analysis was to investigate the accuracy of epicutaneo-caval catheter tip location by comparing ultrasound to conventional radiology. The main question adressed in the research was if it is possible to perform a systematic review and meta-analysis about tip location of epicutaneo-caval catheters (ECCs) and also to give the readers a focus on future research.

   The study is a systematic and meta-analysis review and so, a systematic literature search in multiple databases was performed, resulting in a number of 19 articles that were analyzed and 1094 neonates included. A literature search was performed in PubMed, Eubase, Cochrane library and Web of Science, to evaluate the articles about tip location in ECC using X-Ray and ultrasonography in neonates, updated to 2022. The topic is original and relevant in the field and brings usefull knowledge regarding the subject. A comprehensive search strategy was used. The review methodology was comprehensive with screening and data extraction. When it comes to the methodology used, no specific improvements should be considered from my point of view.

   The conclusions are consistent with the evidence and the arguments presented, and they adress properly to the main question which conducted the analysis.

   The references have been verified and are appropriate for the study. 

    Regarding the figures and tables used in the article, they are very understandable, explicit and easy to be followed and they adress properly for this kind of study, so no other comments regarding this subject are necessary. 

  Regarding the structure and accuracy of the phrases, the manuscript has well structured information, with supported evidence and well structured phrases.

   The manuscript is original and well defined. The results provide an advance in current knowledge. The results are being interpreted appropriately and are significant, as well as the conclusions.

  The study is correctly designed and the analysis is being performed at high standards, so the data are robust enough to draw the conclusion. Surely the paper will attract a wide readership. 

   To conclude, the article is written in a proper way and brings useful information regarding the subject. 

Line 2: epicutaneo-caval, not „epicutaneo-cava”

Line 18: epicutaneo-caval, not „epicutaneo-cava”

Line 20: epicutaneo-caval, not „epicutaneo-cava”

Line 36: „.” after „[3]”

Line 55: „.” after „[3]”

Line 58: „,” after „ECC”

Line 60: „,” after „attempt”

Line 87: „,” after „guideline”

Line 87: „,” after „neonates”

Line 99: „,” before „updated”

Line 124: A total number of 1094 neonates were included, not „Total neonates included were 1094”

Line 242: only 1 space between „.” and „Therefore”

Line 339: „,” after „US”

Line 339: „,” after „nowadays”

Author Response

 The main purpose of the analysis was to investigate the accuracy of epicutaneo-caval catheter tip location by comparing ultrasound to conventional radiology. The main question addressed in the research was if it is possible to perform a systematic review and meta-analysis about tip location of epicutaneo-caval catheters (ECCs) and also to give the readers a focus on future research.

  The study is a systematic and meta-analysis review and so, a systematic literature search in multiple databases was performed, resulting in a number of 19 articles that were analyzed and 1094 neonates included. A literature search was performed in PubMed, Eubase, Cochrane library and Web of Science, to evaluate the articles about tip location in ECC using X-Ray and ultrasonography in neonates, updated to 2022. The topic is original and relevant in the field and brings usefull knowledge regarding the subject. A comprehensive search strategy was used. The review methodology was comprehensive with screening and data extraction. When it comes to the methodology used, no specific improvements should be considered from my point of view.

The conclusions are consistent with the evidence and the arguments presented, and they address properly to the main question which conducted the analysis.

   The references have been verified and are appropriate for the study. 

    Regarding the figures and tables used in the article, they are very understandable, explicit and easy to be followed and they address properly for this kind of study, so no other comments regarding this subject are necessary. 

  Regarding the structure and accuracy of the phrases, the manuscript has well-structured information, with supported evidence and well structured phrases.

 The manuscript is original and well defined. The results provide an advance in current knowledge. The results are being interpreted appropriately and are significant, as well as the conclusions.

  The study is correctly designed and the analysis is being performed at high standards, so the data are robust enough to draw the conclusion. Surely the paper will attract a wide readership. 

 To conclude, the article is written in a proper way and brings useful information regarding the subject. 

Tank you for your comments.

Comments on the Quality of English Language

Line 2: epicutaneo-caval, not „epicutaneo-cava”

Line 18: epicutaneo-caval, not „epicutaneo-cava”

Line 20: epicutaneo-caval, not „epicutaneo-cava”

Line 36: „.” after „[3]”

Line 55: „.” after „[3]”

Line 58: „,” after „ECC”

Line 60: „,” after „attempt”

Line 87: „,” after „guideline”

Line 87: „,” after „neonates”

Line 99: „,” before „updated”

Line 124: A total number of 1094 neonates were included, not „Total neonates included were 1094”

Line 242: only 1 space between „.” and „Therefore”

Line 339: „,” after „US”

Line 339: „,” after „nowadays”

Thank you for your suggestions. We modified the text according to your comment.

Round 2

Reviewer 5 Report

The authors present a revised version of their manuscript on the role of ultrasound in epicutaneo-caval catheter insertion in neonates.

Substantial comments were provided by multiple reviewers and the revision failed to answer most of them. The first is the problem with the term »epicutaneo-caval catheter«. The authors did explain that PICC and ECC are different kind of catheters, however this leads to major inconsistencies in the research. Their literature search included the terms PICC and »peripherally inserted central catheter« - why include this term, when ECC is a different kind of catheter?

I extracted the first cited article that was included in their meta-analysis (Reference #1: A. Jain et al. “The Use of Targeted Neonatal Echocardiography to Confirm Placement of Peripherally Inserted Central Catheters in Neonates,” Am J Perinatol, vol. 29, no. 02, pp. 101–106, Feb. 2012, doi: 10.1055/s- 484 0031-1295649). This article talks about PICCs. There is no hint that only ECCs in the context of what was described in the presented manuscript were evaluated here. How did this article meet the inclusion criteria? I will not check all 19 included articles, as this is authors responsibility, however this is not acceptable.

The aim of the study is still not correctly stated to really describe what the study aimed to do.

The methods were barely revised. A lot of details are still missing. In the methods section it should be explained more in detail about the article selection process. This is the key part of the review articles. Did each author independently do the literature search and review? Did the results match? How were discrepancies managed? How was it assured that the included articles actually fit the criteria? Who performed abstract reading? Who performed whole article reading and data extraction?

What was the quality of the included articles? Did you evaluate that at all(AXIS tool?)? This is also a key part of review meta-analysis.

The discussion was barely revised. Only a single longer paragraph was added – one describing the difference between PICC and ECC – the part that should actually be fitting in the introduction section. The beginning of the discussion remained the same – a copy of information from the introduction. In the discussion you proclaim radiography as an inaccurate method to determine the catheter position, while later you state that it is correct in 76,6% while US is correct in 78% - this is hardly a difference (page 13, line 348). The whole unrelated part of Neo-ECHOTIP protocol is still there.

Overall, the revision was performed very hastily. There are uncountable typos and English language errors in the revised parts. Additionally, the original manuscript was also not improved in the use of the English language.

The quality of English language is poor.

Author Response

The authors present a revised version of their manuscript on the role of ultrasound in epicutaneo-caval catheter insertion in neonates.

Substantial comments were provided by multiple reviewers and the revision failed to answer most of them. The first is the problem with the term »epicutaneo-caval catheter«. The authors did explain that PICC and ECC are different kind of catheters, however this leads to major inconsistencies in the research. Their literature search included the terms PICC and »peripherally inserted central catheter« - why include this term, when ECC is a different kind of catheter?

Thank you for your comment. Since the term ECC is a quite recent terminology introduced by the WoCoVa it seems very reasonable for the authors to include the word PICC in the research. This choice reflects our intention to look for any relevant paper in this field. Of course, as part of our process the papers which do not refer to ECC were excluded. In fact, only studies involving neonates (therefore ECC) were selected for the analysis.

I extracted the first cited article that was included in their meta-analysis (Reference #1: A. Jain et al. “The Use of Targeted Neonatal Echocardiography to Confirm Placement of Peripherally Inserted Central Catheters in Neonates,” Am J Perinatol, vol. 29, no. 02, pp. 101–106, Feb. 2012, doi: 10.1055/s- 484 0031-1295649). This article talks about PICCs. There is no hint that only ECCs in the context of what was described in the presented manuscript were evaluated here. How did this article meet the inclusion criteria? I will not check all 19 included articles, as this is authors responsibility, however this is not acceptable.

Thank you for your comment. Again this paper is about ECC in neonates. The terminology PICC has been used in a wrong way by Jain and coworkers, but this doesn’t mean that their research should be excluded since at the end of the day is about ECC and tip location. We would like again to make clear that the placement of PICC catheter (as intended by the WoCoVA) is not feaseable in neonates since there are no veins suitable for such purpose.

The aim of the study is still not correctly stated to really describe what the study aimed to do.

The aim of the study was stated in line 111. We think it’s clearly stated

The methods were barely revised. A lot of details are still missing. In the methods section it should be explained more in detail about the article selection process. This is the key part of the review articles. Did each author independently do the literature search and review? Did the results match? How were discrepancies managed? How was it assured that the included articles actually fit the criteria? Who performed abstract reading? Who performed whole article reading and data extraction? What was the quality of the included articles? Did you evaluate that at all(AXIS tool?)? This is also a key part of review meta-analysis.

Thank you for your comment. The methods section  was extensively revised.

The discussion was barely revised. Only a single longer paragraph was added – one describing the difference between PICC and ECC – the part that should actually be fitting in the introduction section. The beginning of the discussion remained the same – a copy of information from the introduction. In the discussion you proclaim radiography as an inaccurate method to determine the catheter position, while later you state that it is correct in 76,6% while US is correct in 78% - this is hardly a difference (page 13, line 348). The whole unrelated part of Neo-ECHOTIP protocol is still there.

The discussion was extensively revised. The NeoECHOTIP is very related to the present work and was manteined in the paper. The number 76.6 vs 78 is the number of tip visualised as clearly stated in the paper and not the number of tip in a correct position. We do hope that this new version of the discussion help the reviewer and the reader to understand the results

Overall, the revision was performed very hastily. There are uncountable typos and English language errors in the revised parts. Additionally, the original manuscript was also not improved in the use of the English language.

Thank you for your comment. the text was again revised for the English style.

Reviewer 6 Report

              Regarding the structure and accuracy of the phrases, after the corrections were made, the manuscript has indeed well structured information and the phrases are well designed.

             The manuscript is original and well defined and  the results provide an advance in current knowledge. The results are being interpreted appropriately and are significant and after improving  the writting techniques and the structure of the phrases, it is an article worth reading and the meaning of it is well understandable.  So now, the article is  written in an appropriate way. 

             The data are robust enough and it is easy to follow the conclusions. 

             Surely the paper will attract a wide readership. 

            The English language is now appropriate.

            To conclude, from my point of view, the manuscript has been sufficiently improved to warrant publication

Author Response

Thank you for your positive comments